# Assessment of Screening Tools to Identify Substance Use Disorders Among Adolescents

**DOI:** 10.1001/jamanetworkopen.2023.14422

**Published:** 2023-05-22

**Authors:** Sharon Levy, Melissa Brogna, Machiko Minegishi, Geetha Subramaniam, Jennifer McCormack, Margaret Kline, Eleanor Menzin, Sophie Allende-Richter, Alyssa Fuller, Mitra Lewis, Julia Collins, Zach Hubbard, Shannon G. Mitchell, Roger Weiss, Elissa Weitzman

**Affiliations:** 1 Division of Addiction Medicine, Boston Children’s Hospital, Boston, Massachusetts; 2 Department of Pediatrics, Harvard Medical School, Boston, Massachusetts; 3 Center for Clinical Trials Network, National Institute on Drug Abuse, Bethesda, Maryland; 4 Emmes Company, Rockville, Maryland; 5 Division of General Pediatrics, Boston Children’s Hospital, Boston, Massachusetts; 6 Friends Research Institute, Baltimore, Maryland; 7 Department of Psychiatry, Harvard Medical School, Boston, Massachusetts; 8 Division of Alcohol, Drugs, and Addiction, McLean Hospital, Belmont, Massachusetts; 9 Division of Adolescent and Young Adult Medicine, Boston Children’s Hospital, Boston, Massachusetts; 10 Computational Health Informatics Program, Boston Children’s Hospital, Boston, Massachusetts

## Abstract

**Importance:**

Efficient screening tools that effectively identify substance use disorders (SUDs) among youths are needed.

**Objective:**

To evaluate the psychometric properties of 3 brief substance use screening tools (Screening to Brief Intervention [S2BI]; Brief Screener for Tobacco, Alcohol, and Drugs [BSTAD]; and Tobacco, Alcohol, Prescription Medication, and Other Substances [TAPS]) with adolescents aged 12 to 17 years.

**Design, Setting, and Participants:**

This cross-sectional validation study was conducted from July 1, 2020, to February 28, 2022. Participants aged 12 to 17 years were recruited virtually and in person from 3 health care settings in Massachusetts: (1) an outpatient adolescent SUD treatment program at a pediatric hospital, (2) an adolescent medicine program at a community pediatric practice affiliated with an academic institution, and (3) 1 of 28 participating pediatric primary care practices. Participants were randomly assigned to complete 1 of the 3 electronic screening tools via self-administration, followed by a brief electronic assessment battery and a research assistant–administered diagnostic interview as the criterion standard measure for *Diagnostic and Statistical Manual of Mental Disorders, Fifth Edition* (*DSM-5*) diagnoses of SUDs. Data were analyzed from May 31 to September 13, 2022.

**Main Outcomes and Measures:**

The main outcome was a *DSM-5* diagnosis of tobacco/nicotine, alcohol, or cannabis use disorder as determined by the criterion standard World Mental Health Composite International Diagnostic Interview Substance Abuse Module. Classification accuracy of the 3 substance use screening tools was assessed by examining the agreement between the criterion, using sensitivity and specificity, based on cut points for each tool for use disorder, chosen a priori from previous studies.

**Results:**

This study included 798 adolescents, with a mean (SD) age of 14.6 (1.6) years. The majority of participants identified as female (415 [52.0%]) and were White (524 [65.7%]). High agreement between screening results and the criterion standard measure was observed, with area under the curve values ranging from 0.89 to 1 for nicotine, alcohol, and cannabis use disorders for each of the 3 screening tools.

**Conclusions and Relevance:**

These findings suggest that screening tools that use questions on past-year frequency of use are effective for identifying adolescents with SUDs. Future work could examine whether these tools have differing properties when used with different groups of adolescents in different settings.

## Key Points

**Question:**

Do existing screening tools, such as the Screening to Brief Intervention (S2BI), Brief Screener for Tobacco, Alcohol, and Drugs (BSTAD), and Tobacco, Alcohol, Prescription Medication, and Other Substances (TAPS), accurately identify substance use disorders among youths?

**Findings:**

In this cross-sectional study of 798 adolescents, high agreement was observed between results for the S2BI, BSTAD, and TAPS tools and for the criterion standard measure (a brief electronic assessment battery and a research assistant–administered diagnostic interview). Area under the curve values were near or equal to 1 for nicotine, alcohol, and cannabis use disorders for each of the 3 screening tools.

**Meaning:**

These findings suggest that brief screening tools that measure past-year frequency of use effectively identify substance use disorders among youths.

## Teaser

This cross-sectional study evaluates the psychometric properties of 3 brief screening tools that measure past-year frequency of nicotine/tobacco, alcohol, cannabis, and other drug use and their efficacy in identifying substance use disorders among youths.

## Introduction

Adolescent substance use is an important and modifiable behavioral health concern for youths. A burgeoning evidence base is demonstrating that intervention in pediatric primary care can improve outcomes.^1,2,3^ Clinicians need screening tools that can accurately identify youths with substance use disorders (SUDs) so that they may be triaged appropriately. Several brief screening tools have been developed and validated to identify substance use problems and disorders with adolescent primary care patients. The Screening to Brief Intervention (S2BI),^4^ the Brief Screener for Tobacco, Alcohol, and Drugs (BSTAD),^5^ and the Tobacco, Alcohol, Prescription Medication, and Other Substances (TAPS)^6^ are all brief screening tools that use a question on past-year frequency of use to generate separate risk levels for alcohol, tobacco, and cannabis use disorders. These 3 tools were selected for this study because they have been initially validated, albeit with small, homogeneous, or adult populations.^4,5,6^ The current study was undertaken to replicate and extend the evaluation of (1) the S2BI through a larger sample, (2) the BSTAD through a more diverse sample, and (3) the TAPS in a younger sample.

## Methods

This cross-sectional validation study was conducted with approval and a waiver of parental consent granted by the Boston Children’s Hospital Institutional Review Board. Parental consent was waived in this low-risk study because previous work has demonstrated that a requirement for parental consent biases the sample to a lower-risk population. The study followed the Standards for Reporting of Diagnostic Accuracy (STARD).

### Participant Recruitment

This study was conducted between July 1, 2020, and February 28, 2022. Due to restrictions on medical care facilities in response to the COVID-19 pandemic, the study was conducted entirely virtually from July 1, 2020, through February 28, 2021; we used a hybrid model from March 1, 2021, through February 28, 2022.

Participants aged 12 to 17 years were recruited virtually and in person from 3 health care settings in Massachusetts: (1) an outpatient adolescent SUD treatment program at a pediatric hospital, (2) an adolescent medicine program at a community pediatric practice affiliated with an academic institution, and (3) 1 of 28 participating pediatric primary care practices. The enrollment of younger adolescents (aged 12-13 years) was capped at 200 because substance use is less common in this age group.^7^

Recruitment strategies evolved in response to restrictions resulting from the COVID-19 pandemic. We used a combination of virtual and in-person strategies, including direct referral from a primary care physician during a virtual health care visit, patient portal messaging, posters and postcards with links to study information, telephone calls made to patients and parents, and in-person recruitment at the study sites. Each recruitment strategy is described in detail in eFigure 1 in Supplement 1.

We defined “attempted to contact” as any patient who (1) entered a videoconference room, (2) provided contact information (including emailed interest or called or texted the study phone number), (3) received a call from a research assistant (RA), (4) received study information in a videoconference chat during an appointment, or (5) was scheduled for an in-person appointment on a day when a study RA was present in the clinic. Of the 2840 potential participants whom we attempted to contact, approximately 1411 had spoken directly with an RA and were considered invited to participate in the study; the remaining 1429 could not be reached (Figure). Of the 1411 invited patients, 364 (25.8%) were not interested and 1047 (74.2%) completed an anonymous eligibility screener.

**Figure.**
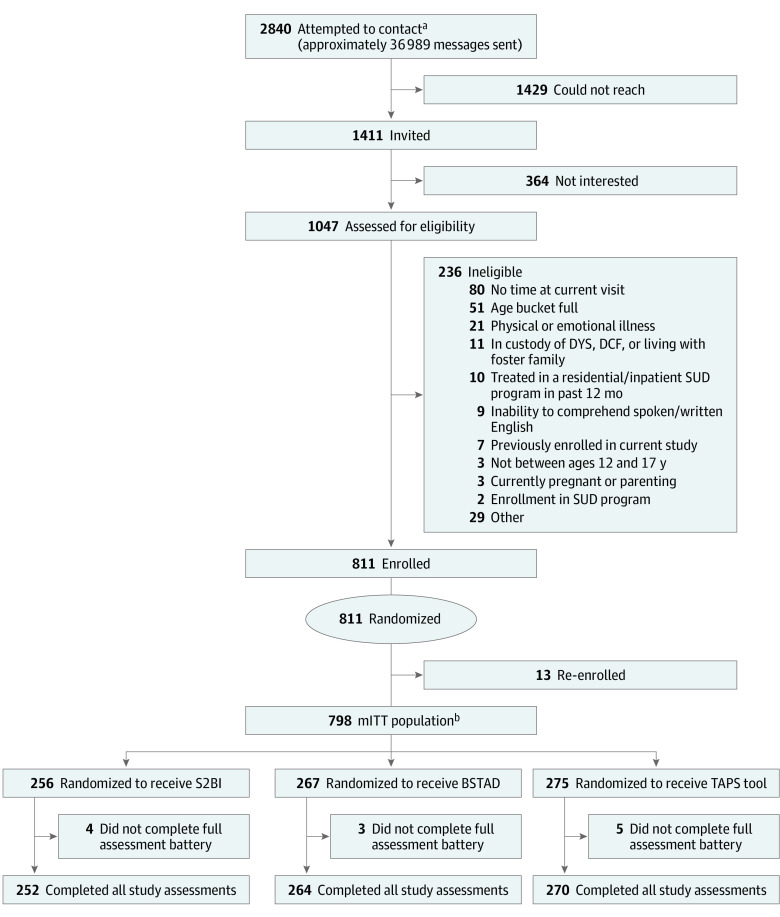
Study Flow Diagram BSTAD indicates Brief Screener for Tobacco, Alcohol, and Drugs; DCF, Massachusetts Department of Children and Families; DYS, Massachusetts Department of Youth Services; mITT, modified intention to treat; S2BI, Screening to Brief Intervention; SUD, substance use disorder; TAPS, Tobacco, Alcohol, Prescription Medication, and Other Substances. ^a^“Attempted to contact” was defined as any patient who (1) entered a videoconference room, (2) provided contact information (including emailed interest or called or texted the study phone number), (3) received a call from a research assistant, (4) received study information in a videoconference chat during an appointment, or (5) was scheduled for an in-person appointment on a day when a study research assistant was present in the clinic. ^b^The miTT population consisted of those who completed the study once and whose data were used to analyze alcohol, cannabis, and tobacco use. All mITT participants were considered in the primary analysis but had to have both screening tool question(s) and a World Mental Health Composite International Diagnostic Interview Substance Abuse Module score for a substance (tobacco/nicotine, alcohol, or cannabis) to be able to be used in calculating the psychometric properties.

### Exclusion Criteria

Patients were excluded if they were unable to understand English, were physically or emotionally unwell at the time of recruitment, had been admitted to a residential or inpatient SUD treatment program within the past 12 months (because we believed this might affect their response to screening questions), were currently in the custody of the Massachusetts Department of Youth Services or the Department of Children and Families, were living with a foster family, or were pregnant or parenting. Patients at primary care sites who were currently enrolled in an SUD treatment program at the time of recruitment were also excluded. Based on these criteria, 236 of the 1047 patients (22.5%) were ineligible; the remaining 811 patients provided assent and were enrolled in this study. However, 13 screening results were later linked to 5 single individuals and thus eliminated from the overall sample. Of the 798 randomized participants, 12 (1.5%) did not complete the study, leaving a total of 786 who completed all study assessments (Figure). All participants received a $10 gift card upon study completion.

###  Screening Tools and Assessment Battery

Participants were randomized to receive 1 of 3 screening tools—the S2BI (n = 256), BSTAD (n = 267), or TAPS (n = 275)—using a randomization scheme balanced for age (12-13 years vs 14-17 years), sex (male vs female), and clinical setting to ensure baseline comparability of the cohorts assigned to the 3 tools. Screening tool assignments were randomly generated and assigned using a permuted block design with blocks of varying sizes within strata. We administered 1 tool to each participant to avoid the response to an initial screen affecting the response to a subsequent one.

Participants self-administered both the screening tool electronically according to their randomization group and a brief electronic assessment battery. An RA then administered a modified version of the World Mental Health Composite International Diagnostic Interview Substance Abuse Module (WMH-CIDI-SAM).^8^ All participants in the SUD treatment program completed the screening tool and assessment battery before their initial appointment. The screening tools are described in detail in the eMethods in Supplement 1.

The self-administered assessment battery included 7 demographic items (age, sex, gender, race, ethnicity, number of parents or caregivers at home, and highest level of education completed by parents or caregivers), screens for depression (2-question Patient Health Questionnaire [PHQ-2]^9^) and anxiety (2-item Generalized Anxiety Disorder [GAD-2] Scale^10^), and 2 questions to screen for attention-deficit/hyperactivity disorder (ADHD) or attention-deficit disorder (ADD). The WMH-CIDI-SAM^8^ was administered by a trained RA. Participants self-reported their race (Asian, Black or African American [hereinafter Black], White, other, or multiple races) and ethnicity (Hispanic or Latino [hereinafter Hispanic] or non-Hispanic). We collected data on race and ethnicity and planned a priori to perform exploratory subgroup analyses on different demographics, including sex, race and ethnicity, and age, as required by the 2016 National Institutes of Health guidelines on inclusion.^11^ Participant responses were mapped to SUD diagnostic criteria in the *Diagnostic and Statistical Manual of Mental Disorders, Fifth Edition* (*DSM-5*).^12^ The criterion for interpersonal problems was met if both WMH-CIDI-SAM items were satisfied, and the criteria for uncontrolled escalation of use and withdrawal symptoms were met if either WMH-CIDI-SAM item was satisfied. Participants who endorsed 2 or more criteria were considered to meet criteria for a SUD (eTable 1 in Supplement 1).

### Statistical Analysis

All 798 participants in the modified intention-to-treat (mITT) population were considered in the primary descriptive analysis but had to have both screening tool question(s) and a criterion standard measure for a substance (tobacco/nicotine, alcohol, and cannabis) to be used in calculating the psychometric properties. Details of randomization, screening, and criterion measure responses for tobacco/nicotine, alcohol, or cannabis use disorders are summarized in STARD flow charts in eFigures 2, 3, and 4 in Supplement 1, respectively.

Baseline characteristics were described and compared by study site, using analysis of variance or the Kruskal-Wallis test for continuous variables and the Fisher exact or χ^2^ test for categorical variables, as appropriate. Responses on the S2BI, BSTAD, and TAPS tools were compared with *DSM-5* criteria as measured by the modified WMH-CIDI-SAM, which was the criterion measure for SUD. Sensitivity and specificity were evaluated. Cut points were chosen a priori based on previous studies.^4,5,6^ To evaluate the level of classification accuracy for each measure for each screening tool for tobacco/nicotine, alcohol, and cannabis use, statistical analyses examined the level of agreement between different measures of substance use. Receiver operating characteristic (ROC) curves were plotted to estimate concordance for tobacco/nicotine, alcohol, and cannabis use disorders measured by the WMH-CIDI-SAM for each of the 3 screening tools. Area under the curve values indicated levels of agreement (>0.7, moderate; >0.8, good; and >0.9, high).^13^

Analyses were conducted using SAS, version 9.4 (SAS Institute Inc). Statistical significance was set at *P* < .05, and tests were 2 tailed. Data were analyzed from May 31 to September 13, 2022.

## Results

### Participant Description

This cross-sectional study comprised 798 participants, with a mean (SD) age of 14.6 (1.6) years (Table 1). For self-reported gender, a total of 415 participants (52.0%) identified as female, 365 (45.7%) as male, and 17 (2.1%) as other gender; 1 participant (0.1%) preferred not to answer. With regard to self-reported race, 69 participants (8.6%) were Asian, 61 (7.6%) were Black, 524 (65.7%) were White, 50 (6.3%) were of other race, and 53 (6.6%) were of multiple races; 41 participants (5.1%) answered unknown, refused to answer, or had missing data. With regard to self-reported ethnicity, 122 participants (15.3%) identified as Hispanic and 645 (80.8%) as non-Hispanic; 31 participants (3.9%) answered unknown, refused to answer, or had missing data.

**Table 1.** Sociodemographics and Health Characteristics by Study Site^a^

Characteristic	Total population (N = 798)^c^	Study site^b^			*P* value
		SUD treatment program (n = 41 [5.1%])	Adolescent medicine program (n = 84 [10.5%])	Primary care (n = 673 [84.3%])	
Sex assigned at birth					
Male	364 (45.6)	28 (68.3)	30 (35.7)	306 (45.5)	.007
Female	433 (54.3)	13 (31.7)	54 (64.3)	366 (54.4)	
Unknown	1 (0.1)	0	0	1 (0.1)	
Gender^d^					
Male	365 (45.7)	28 (68.3)	29 (34.5)	308 (45.8)	<.001
Female	415 (52.0)	11 (26.8)	53 (63.1)	351 (52.2)	
Other	17 (2.1)	2 (4.9)	1 (1.2)	14 (2.1)	
Preferred not to answer	1 (0.1)	0	1 (1.2)	0	
Age, y					
Mean (SD)	14.6 (1.6)	15.7 (1.3)	15.8 (1.3)	14.4 (1.5)	<.001
12-13	199 (24.9)	3 (7.3)	4 (4.8)	192 (28.5)	<.001
14-17	599 (75.1)	38 (92.7)	80 (95.2)	481 (71.5)	
Ethnicity^d^					
Hispanic or Latino	122 (15.3)	4 (9.8)	72 (85.7)	46 (6.8)	<.001
Non-Hispanic or non-Latino	645 (80.8)	33 (80.5)	10 (11.9)	602 (89.5)	
Unknown, refused to answer, or missing	31 (3.9)	4 (9.8)	2 (2.4)	25 (3.7)	
Race^d^					
Asian	69 (8.6)	0	0	69 (10.3)	<.001
Black or African American	61 (7.6)	6 (14.6)	32 (38.1)	23 (3.4)	
White	524 (65.7)	29 (70.7)	10 (11.9)	485 (72.1)	
Other^e^	50 (6.3)	1 (2.4)	24 (28.6)	25 (3.7)	
Multiple races	53 (6.6)	2 (4.9)	5 (6.0)	46 (6.8)	
Unknown, refused to answer, or missing	41 (5.1)	3 (7.3)	13 (15.5)	25 (3.7)	
No. of parents or caregivers living with participant					
0 or 1	121 (15.2)	7 (17.1)	44 (52.4)	70 (10.4)	<.001
2	637 (79.8)	31 (75.6)	34 (40.5)	572 (85.0)	
>2	25 (3.1)	0	4 (4.8)	21 (3.1)	
Missing	15 (1.9)	3 (7.3)	2 (2.4)	10 (1.5)	
Parental or caregiver education^f^					
Less than college^g^	117 (14.7)	7 (17.1)	49 (58.3)	61 (9.1)	<.001
College or more^h^	564 (70.7)	27 (65.9)	11 (13.1)	526 (78.2)	
Unknown	79 (9.9)	4 (9.8)	16 (19.0)	59 (8.8)	
Missing	38 (4.8)	3 (7.3)	8 (9.5)	27 (4.0)	
GAD-2 score^i^					
Negative (0-2)	646 (81.0)	29 (70.7)	69 (82.1)	548 (81.4)	.11
Positive (≥3)	134 (16.8)	9 (22.0)	12 (14.3)	113 (16.8)	
Missing	18 (2.3)	3 (7.3)	3 (3.6)	12 (1.8)	
PHQ-2 score^j^					
Negative (0-2)	681 (85.3)	29 (70.7)	67 (79.8)	585 (86.9)	.01
Positive (≥3)	105 (13.2)	10 (24.4)	14 (16.7)	81 (12.0)	
Missing	12 (1.5)	2 (4.9)	3 (3.6)	7 (1.0)	
ADD or ADHD^k^					
No	638 (79.9)	22 (53.7)	68 (81.0)	548 (81.4)	<.001
Yes	149 (18.7)	17 (41.5)	14 (16.7)	118 (17.5)	
Missing	11 (1.4)	2 (4.9)	2 (2.4)	7 (1.0)	

Abbreviations: ADD, attention-deficit disorder; ADHD, attention-deficit/hyperactivity disorder; GAD, Generalized Anxiety Disorder Scale; PHQ, Patient Health Questionnaire; SUD, substance use disorder.

^a^ Unless indicated otherwise, values are presented as No. (%) of participants.

^b^ Study sites comprised the following, all in Massachusetts: (1) an outpatient adolescent SUD treatment program at a pediatric hospital, (2) an adolescent medicine program at a community pediatric practice affiliated with an academic institution, and (3) 28 participating pediatric primary care practices.

^c^ A total of 798 participants were enrolled but 12 terminated participation early.

^d^ Gender, race, and ethnicity were self-reported.

^e^ This category was created because the samples were small and includes American Indian or Alaska Native or Native Hawaiian or other Pacific Islander.

^f^ Participants were asked: “Of the parent(s)/caregiver(s) who live with you at home, what is the highest level of education he/she has completed?”

^g^ Responses included (1) grade 12 or less or high-school graduate or (2) general educational development test, high-school equivalency test, some college, associate degree, or technical school training.

^h^ This category includes an undergraduate (bachelor) degree or graduate or greater (master, doctorate, etc) degree.

^i^ The GAD-2 uses the first 2 questions of the 7-item GAD scale. Scores range from 0 to 6, with higher scores indicating greater likelihood of generalized anxiety; scores of 3 or greater suggest that generalized anxiety disorder is likely.

^j^ The PHQ-2 uses the first 2 questions of the 9-item PHQ. Scores range from 0 to 6, with higher scores indicating greater likelihood of depression; scores of 3 or greater suggest that a major depressive disorder is likely.

^k^ Participants were asked: “Has a doctor or health care provider ever told you that you have ADD or ADHD? In the past 12 months, have you been prescribed medication for ADD or ADHD?”

The majority of participants (662 [83.0%]) lived in a household with 2 or more caregivers and reported the highest level of caregiver education as a bachelor degree or higher as (564 [70.7%]). There were 134 participants (16.8%) and 105 participants (13.2%) with a score of 3 or greater on the GAD-2 and the PHQ-2, respectively; 149 participants (18.7%) had either been diagnosed with ADHD or ADD by a health care clinician or had received a prescription medication to treat ADHD or ADD.

Sociodemographic characteristics and mental health status of participants differed substantially among the 3 sites, reflecting the differences in patient populations (Table 1). No differences between sociodemographic characteristics or mental health diagnoses by screening tool were observed. The prevalence of SUD diagnoses differed by study site and is presented in eTable 2 in Supplement 1.

### Substance Use Disclosure by Screening Tool

Among the participants recruited from adolescent medicine or primary care, 20 (8.3%), 5 (2.0%), and 11 (4.2%; *P* = .003) individuals disclosed prescription medication use and 65 (27.1%), 47 (18.7%), and 51 (19.6%; *P* = .046) disclosed any substance use on the S2BI, BSTAD, and TAPS tools, respectively (Table 2). Disclosure rates among all randomized participants for other substances are presented in eTables 3 and 4 in Supplement 1.

**Table 2.** Subgroup Analyses of Disclosure of Any Past 12-Month Substance Use Among Primary Care and Adolescent Medicine Patient Groups by Screening Tool^a^

	Subgroup (n = 752)	Screening tool			*P* value
		S2BI (n = 240 [31.9%])^b^	BSTAD (n = 252 [33.5%])^c^	TAPS (n = 260 [34.6%])^d^	
Alcohol use in past 12 mo					
Any	113 (15.0)	42 (17.5)	36 (14.3)	35 (13.5)	.04
Never	630 (83.8)	198 (82.5)	209 (82.9)	223 (85.8)	
Missing	9 (1.2)	0	7 (2.8)	2 (0.8)	
Cannabis use in past 12 mo^e^					
Any	41 (5.5)	18 (7.5)	23 (9.1)	NA	NA
Never	450 (59.8)	222 (92.5)	228 (90.5)	NA	
Missing or not asked	261 (34.7)	0	1 (0.4)	260 (100)	
Tobacco or nicotine use in past 12 mo					
Any	52 (6.9)	19 (7.9)	12 (4.8)	21 (8.1)	.12
Never	694 (92.3)	219 (91.2)	240 (95.2)	235 (90.4)	
Missing	6 (0.8)	2 (0.8)	0	4 (1.5)	
Prescription medication use in past 12 mo					
Any	36 (4.8)	20 (8.3)	5 (2.0)	11 (4.2)	.003
Never	711 (94.5)	218 (90.8)	247 (98.0)	246 (94.6)	
Missing	5 (0.7)	2 (0.8)	0	3 (1.2)	
Other substance use in past 12 mo^e^					
Any	29 (3.9)	6 (2.5)	3 (1.2)	20 (7.7)	NA
Never	716 (95.2)	234 (97.5)	248 (98.4)	234 (90.0)	
Missing	7 (0.9)	0	1 (0.4)	6 (2.3)	
Disclosure (self-report) of any use of alcohol, cannabis, prescription medication, or other substance in past 12 mo					
Self-report	163 (21.7)	65 (27.1)	47 (18.7)	51 (19.6)	.046
No self-report	589 (78.3)	175 (72.9)	205 (81.3)	209 (80.4)	

Abbreviations: BSTAD, Brief Screener for Tobacco, Alcohol, and Drugs; NA, not applicable; S2BI, Screening to Brief Intervention; TAPS, Tobacco, Alcohol, Prescription Medication, and Other Substances.

^a^ Unless indicated otherwise, values are presented as No. (%) of participants.

^b^ Among the 242 participants randomized to S2BI screening, 2 did not receive it. The S2BI assesses past 12-month use of tobacco, alcohol, cannabis, prescription medication, e-cigarette, illegal drugs, inhalants, and herbs or synthetic drugs.

^c^ Among the 253 participants randomized to BSTAD screening, 1 did not receive it. The BSTAD assesses past 12-month use of nicotine/tobacco, alcohol, cannabis, prescription medications, and illegal drugs.

^d^ Among the 262 participants randomized to TAPS screening, 2 did not complete it. The TAPS screening assesses past 12-month use of tobacco, alcohol, illegal drugs (including cannabis), and prescription medications.

^e^ A question on cannabis use in the past 12 months was included in the aggregated “illegal drugs” question in the TAPS. No tests were performed for these items due to lack of comparability. Column percentages are shown. χ^2^ and Fisher exact tests were used to assess the associations (if a difference between observed data and expected data was observed); missing responses were not included in the assessment.

In a subgroup analysis, we assessed the prevalence of disclosure of any past 12-month substance use, using the total subgroup of 752 participants from adolescent medicine and primary care who completed 1 of the 3 screening tools (S2BI, BSTAD, and TAPS). Since the rates of self-disclosure of past 12-month substance use were near universal among patients in the SUD treatment program, they were excluded from this exploratory analysis (Table 2).

### Screening Tool Performance for Identifying SUDs

#### Tobacco/Nicotine

Sensitivity of the S2BI, BSTAD, and TAPS for identifying tobacco/nicotine use disorders at the specified cutoffs was 0.89 (95% CI, 0.52-1.00), 1.00 (95% CI, 0.77-1.00), and 0.63 (95% CI, 0.24-0.91), respectively (Table 3). Specificity of the S2BI, BSTAD, and TAPS tools for identifying tobacco/nicotine use disorders at the specified cutoffs was 0.97 (95% CI, 0.94-0.99), 0.98 (95% CI, 0.95-0.99), and 1.00 (95% CI, 0.99-1.00), respectively (Table 3). Estimated agreement (SE) from the ROC curves was high for each tool (S2BI: 0.99 [0.01]; BSTAD: 1.00 [0.003]; and TAPS: 0.99 [0.01]) (eFigure 5 in Supplement 1).

**Table 3.** Performance of the S2BI, BSTAD, and TAPS Screening Tools vs the WMH-CIDI-SAM for Identifying Substance Use Disorders (SUDs) in the Modified Intention-to-Treat Population

	No. of true positive	No. of false positive	No. of true negative	No. of false negative	Sensitivity (95% CI)	Specificity (95% CI)	PPV (95% CI)	NPV (95% CI)	Positive LR (95% CI)	Negative LR (95% CI)
**S2BI**										
Risk level: SUDs (WMH-CIDI-SAM ≥2)										
Tobacco/nicotine (cutoff: monthly or more)	8	8	234	1	0.89 (0.52-1.00)	0.97 (0.94-0.99)	0.50 (0.25-0.75)	1.00 (0.98-1.00)	26.89 (13.09-55.21)	0.11 (0.02-0.73)
Alcohol (cutoff: monthly or more)	2	12	237	2	0.50 (0.07-0.93)	0.95 (0.92-0.97)	0.14 (0.02-0.43)	0.99 (0.97-1.00)	10.37 (3.37-31.95)	0.53 (0.20-1.40)
Cannabis (cutoff: monthly or more)	12	6	234	1	0.92 (0.64-1.00)	0.98 (0.95-0.99)	0.67 (0.41-0.87)	1.00 (0.98-1.00)	36.92 (16.50-82.63)	0.08 (0.01-0.52)
**BSTAD**										
Risk level: SUDs (WMH-CIDI-SAM ≥2)										
Tobacco/nicotine (cutoff: ≥6 d)	14	6	246	0	1.00 (0.77-1.00)	0.98 (0.95-0.99)	0.70 (0.46-0.88)	1.00 (0.99-1.00)	42.00 (19.05-92.60)	NA^a^
Alcohol (cutoff: ≥2 d)	5	30	223	0	1.00 (0.48-1.00)	0.88 (0.84-0.92)	0.14 (0.05-0.30)	1.00 (0.98-1.00)	8.43 (6.03-11.80)	NA^a^
Cannabis (cutoff: ≥2 d)	17	14	231	2	0.89 (0.67-0.99)	0.94 (0.91-0.97)	0.55 (0.36-0.73)	0.99 (0.97-1.00)	15.66 (9.20-26.64)	0.11 (0.03-0.41)
**TAPS**										
Risk level: SUDs (WMH-CIDI-SAM ≥2)										
Tobacco/nicotine (cutoff: ≥2)	5	0	256	3	0.63 (0.24-0.91)	1.00 (0.99-1.00)	1.00 (0.48-1.00)	0.99 (0.97-1.00)	NA^a^	0.38 (0.15-0.92)
Alcohol (cutoff: ≥2)	7	17	242	2	0.78 (0.40-0.97)	0.93 (0.90-0.96)	0.29 (0.13-0.51)	0.99 (0.97-1.00)	11.85 (6.65-21.10)	0.24 (0.07-0.81)
Cannabis (cutoff: ≥2)	9	1	253	3	0.75 (0.43-0.95)	1.00 (0.98-1.00)	0.90 (0.55-1.00)	0.99 (0.97-1.00)	190.50 (26.22-1384.20)	0.25 (0.09-0.67)

Abbreviations: BSTAD, Brief Screener for Tobacco, Alcohol, and Drugs; LR, likelihood ratio; NA, not appliable; NPV, negative predictive value; PPV, positive predictive value; S2BI, Screening to Brief Intervention; TAPS, Tobacco, Alcohol, Prescription Medication, and Other Substances; WMH-CIDI-SAM, World Mental Health Composite International Diagnostic Interview Substance Abuse Module.

^a^ Estimates and CIs not able to be calculated due to 0 counts or division by 0 are denoted with NA.

#### Alcohol

Sensitivity of the S2BI, BSTAD, and TAPS for identifying alcohol use disorders at the specified cutoffs was 0.50 (95% CI, 0.07-0.93), 1.00 (95% CI, 0.48-1.00), and 0.78 (95% CI, 0.40-0.97), respectively (Table 3). Specificity of the S2BI, BSTAD, and TAPS for identifying alcohol use disorders at the specified cutoffs was 0.95 (95% CI, 0.92-0.97), 0.88 (95% CI, 0.84-0.92), and 0.93 (95% CI, 0.90-0.96), respectively (Table 3). Estimated ROC curve agreement (SE) was high for the S2BI (0.97 [0.02]) and BSTAD (0.93 [0.02]) and good for the TAPS (0.89 [0.09]) (eFigure 6 in Supplement 1).

#### Cannabis

Sensitivity of the S2BI, BSTAD, and TAPS for identifying cannabis use disorders at the specified cutoffs was 0.92 (95% CI, 0.64-1.00), 0.89 (95% CI, 0.67-0.99), and 0.75 (95% CI, 0.43-0.95), respectively (Table 3). Specificity of the S2BI, BSTAD, and TAPS for identifying cannabis use disorders at the specified cutoffs was 0.98 (95% CI, 0.95-0.99), 0.94 (95% CI, 0.91-0.97), and 1.00 (95% CI, 0.98-1.00), respectively (Table 3). Estimated ROC curve agreement (SE) was high for each tool (S2BI: 0.98 [0.01]; BSTAD: 0.99 [0.01]; and TAPS: 0.95 [0.05]) (eFigure 7 in Supplement 1). The results were similar when SUD treatment program participants were omitted from the analysis (eTable 5 in Supplement 1).

## Discussion

In this cross-sectional study, we observed that the S2BI, BSTAD, and TAPS screening tools all had adequate psychometric properties for identifying tobacco/nicotine, alcohol, and cannabis use disorders among adolescents at the recommended cut points. Point estimates of sensitivity and specificity were generally high, and there were no notable differences in performance across the 3 tools for any measure or any substance. Our findings confirm that brief screening tools that ask about the past-year frequency of use are useful for identifying adolescents with SUDs.

The TAPS, which comprises past-use frequency questions combined with questions about problems, contains more questions and takes longer to administer than either the S2BI or BSTAD, yet the results of this study suggest that the psychometric properties were similar. Because our findings suggest that the extra questions did not appear to improve performance, we recommend the shorter tools for widespread implementation in pediatric care settings. However, our findings also suggest that the TAPS may be useful in settings that provide care for patients in both pediatric and adult age ranges in which a single tool may simplify clinical protocols.

Rates of substance use disclosure and prescription medication use were higher among participants screened with the S2BI compared with the other tools. This finding may be of importance because previous studies have found lower rates of substance use disclosure in clinical samples compared with research trials.^14^ A tool that engenders greater levels of disclosure may allow for early identification of substance use. For youths, early initiation of alcohol, cannabis, or tobacco or nicotine use is associated with a greater risk of SUDs,^15^ use of illicit drugs,^16^ misuse of opioids, and opioid use disorders.^17,18,19^ Early identification of substance use and early intervention is recommended by the American Academy of Pediatrics^20^ and other professional organizations,^21^ because even youths who do not have an SUD may be harmed by sporadic substance use and evidence suggests that brief interventions to reduce substance use may be effective in this population.^1,22,23,24^ This is particularly so given the rising rates of lethal opioid overdose,^25^ including among youths who do not have an opioid use disorder. Brief screening tools that encourage disclosure about prescription medication use may allow primary care clinicians to identify “at-risk” youths, allowing them to intervene early in this high-risk behavior.

### Limitations

Several limitations of this cross-sectional study should be noted. Participants were heterogeneous regarding gender, race, and ethnicity, and our practices were spread across the state of Massachusetts, including in urban, suburban, and rural settings. Nonetheless, participants were predominantly White, non-Hispanic, and from higher-income backgrounds recruited entirely from Massachusetts, which may limit generalizability. Furthermore, we note that participants were recruited during the COVID-19 pandemic, which may have affected the results. However, our findings were similar to studies with these tools conducted before 2020, including a study conducted in a different geographic region.^5^ Future work with participants recruited from a wider geographic region and from more diverse populations, including youths who do not speak English, could further refine these findings. The proportion of participants reporting past-year substance use was lower than anticipated based on prior research^4^ and may be related to recruitment strategies. Most patients were recruited through a message sent to an online health portal, which was received by both patients and parents. While this study was conducted with a waiver of parental consent, it is possible that some patients were alerted to the opportunity of study participation through their parents; it has previously been demonstrated that adolescents who are recruited to studies that ask about SUDs with their parents’ knowledge have a lower risk for these disorders compared with studies with no parent involvement.^26^ Furthermore, adolescents who were monitoring emails more closely and who found the study invitation independently may be more involved in school activities, a known protective factor against adolescent substance use.^27^ The secular trend toward decreased substance use among adolescents during the COVID-19 pandemic lockdown may also have contributed to lower rates of substance use.^28^ The screening tools and the criterion standard interview used rely on self-report of substance use, which may be affected by underreporting. We used the WHM-CIDI-SAM as the criterion standard for identifying SUDs, which is accepted in research communities, although this tool has not been validated in youths. Low rates of SUDs led to lower precision and wider CIs. It is possible that a larger or higher-risk study population may have helped to identify clinically relevant differences in performance among the tools across settings.

## Conclusions

The findings of this study suggest that the S2BI, BSTAD, and TAPS tools all have adequate psychometric properties for screening adolescents for SUDs in general primary care settings and can be recommended for substance use screening of adolescent patients. Future work could examine whether these tools have differing properties when used with different groups of adolescents in different settings.

## Notes

Supplement 1. **eMethods.** Screening Tools **eFigure 1.** Description of Recruitment Methods **eFigure 2.** STARD Flow Diagram for Tobacco/Nicotine Use Disorder (TUD) **eFigure 3.** STARD Flow Diagram for Alcohol Use Disorder (AUD) **eFigure 4.** STARD Flow Diagram for Cannabis Use Disorder (CUD) **eFigure 5.** ROC Curves for S2BI, BSTAD, and TAPS Tool Screeners and Tobacco/Nicotine Use mITT Population **eFigure 6.** ROC Curves for S2BI, BSTAD, and TAPS Tool Screeners and Alcohol Use mITT Population **eFigure 7.** ROC Curves for S2BI, BSTAD, and TAPS Tool Screeners and Cannabis Use mITT Population **eTable 1.** Prevalence of Substance Use Disorder on the Criterion Measure (CIDI) Among mITT Participants (N=798) **eTable 2.** Prevalence of Substance Use Disorders by Study Site, Based on the Criterion (CIDI) Among mITT Participants (N=798) **eTable 3.** Disclosure of Other Past 12-Month Substance Use Among the Participants Randomized to S2BI/BSTAD Screeners (n=523) **eTable 4.** Disclosure of Any Past 3-Month Substance Use Among the Participants Randomized to the TAPS Screener (n=275) **eTable 5.** Performance of S2BI, BSTAD, and TAPS Tool Screeners vs CIDI for Identifying Substance Use Disorder, Excluding SUDTP Population
eReferences

Supplement 2.
Data Sharing Statement
