# Supplementary material for: Assessment of Screening Tools to Identify Substance Use Disorders Among Adolescents
Source: JAMA Netw Open. 2023 May 22;6(5):e2314422. doi: 10.1001/jamanetworkopen.2023.14422 (PMC10203888; doi:10.1001/jamanetworkopen.2023.14422)
Supplement: Supplement 1. — eMethods. Screening Tools eFigure 1. Description of Recruitment Methods eFigure 2. STARD Flow Diagram for Tobacco/Nicotine Use Disorder (TUD) eFigure 3. STARD Flow Diagram for Alcohol Use Disorder (AUD) eFigure 4. STARD Flow Diagram for Cannabis Use Disorder (CUD) eFigure 5. ROC Curves for S2BI, BSTAD, and TAPS Tool Screeners and Tobacco/Nicotine Use mITT Population eFigure 6. ROC Curves for S2BI, BSTAD, and TAPS Tool Screeners and Alcohol Use mITT Population eFigure 7. ROC Curves for S2BI, BSTAD, and TAPS Tool Screeners and Cannabis Use mITT Population eTable 1. Prevalence of Substance Use Disorder on the Criterion Measure (CIDI) Among mITT Participants (N=798) eTable 2. Prevalence of Substance Use Disorders by Study Site, Based on the Criterion (CIDI) Among mITT Participants (N=798) eTable 3. Disclosure of Other Past 12-Month Substance Use Among the Participants Randomized to S2BI/BSTAD Screeners (n=523) eTable 4. Disclosure of Any Past 3-Month Substance Use Among the Participants Randomized to the TAPS Screener (n=275) eTable 5. Performance of S2BI, BSTAD, and TAPS Tool Screeners vs CIDI for Identifying Substance Use Disorder, Excluding SUDTP Population eReferences [file jamanetwopen-e2314422-s001.pdf]

Supplementary Online Content

Levy S, Brogna M, Minegishi M, et al. Assessment of screening tools to identify substance use disorders among adolescents. *JAMA Netw Open*. 2023;6(5):e2314422. doi:10.1001/jamanetworkopen.2023.14422

eMethods. Screening Tools

eFigure 1. Description of Recruitment Methods

eFigure 2. STARD Flow Diagram for Tobacco/Nicotine Use Disorder (TUD)

eFigure 3. STARD Flow Diagram for Alcohol Use Disorder (AUD)

eFigure 4. STARD Flow Diagram for Cannabis Use Disorder (CUD)

eFigure 5. ROC Curves for S2BI, BSTAD, and TAPS Tool Screeners and Tobacco/Nicotine Use mITT Population

eFigure 6. ROC Curves for S2BI, BSTAD, and TAPS Tool Screeners and Alcohol Use mITT Population

eFigure 7. ROC Curves for S2BI, BSTAD, and TAPS Tool Screeners and Cannabis Use mITT Population

eTable 1. Prevalence of Substance Use Disorder on the Criterion Measure (CIDI) Among mITT Participants (N=798)

eTable 2. Prevalence of Substance Use Disorders by Study Site, Based on the Criterion (CIDI) Among mITT Participants (N=798)

eTable 3. Disclosure of Other Past 12-Month Substance Use Among the Participants Randomized to S2BI/BSTAD Screeners (n=523)

eTable 4. Disclosure of Any Past 3-Month Substance Use Among the Participants Randomized to the TAPS Screener (n=275)

eTable 5. Performance of S2BI, BSTAD, and TAPS Tool Screeners vs CIDI for Identifying Substance Use Disorder, Excluding SUDTP Population

eReferences

This supplementary material has been provided by the authors to give readers additional information about their work.

© 2023 Levy S et al. *JAMA Network Open*.

eMethods. Screening Tools

Screening to Brief Intervention (S2BI)[1,2]
The Screening to Brief Intervention (S2BI) tool is a 7-question screen that uses a stem question (In the past year, how many times have you used [X]) and forced response options (none, once or twice, monthly, and weekly or more) to identify the frequency of past-year use of tobacco/nicotine, alcohol, cannabis, and 4 other classes of substances most commonly used by adolescents (prescription drugs that were not prescribed for you, inhalants, illegal drugs, herbs or synthetic drugs). S2BI screener results are coded as follows: “missing”, “never”, “once or twice”, “monthly”, and “weekly or more”. The cut point of “monthly” or “weekly or more” was used to identify substance use disorders.

Brief Screener for Tobacco, Alcohol and Drugs (BSTAD)[3]
The Brief Screener for Tobacco, Alcohol and Drugs (BSTAD) is an 11-question screen that asks about the number of days of use of tobacco/nicotine, alcohol, and cannabis in the past year, in accordance with National Institute on Alcohol Abuse and Alcoholism (NIAAA)’s convention. The screener also includes questions about past-year frequency of use (number of days) of illicit substances and misused prescription medications. The BSTAD screen results are presented numerically and categorically (responses range from 0-365 days: “missing,” “0 days,” “1 day,” “2 days,” “3-5 days,” “≥ 6 days”). The cut points of “≥ 6 days” for tobacco/nicotine and “≥ 2 days” for alcohol and cannabis were used to identify SUD.

Tobacco, Alcohol, Prescription medications, and other Substance (TAPS) Tool[4]
The Tobacco, Alcohol, Prescription medications, and other Substance (TAPS) Tool is a two-part questionnaire. The TAPS-1 is the screening component that asks about the frequency of past year use of tobacco/nicotine, alcohol, illicit drugs, and misuse of prescription medications with 5 response categories ranging from “never” to “daily or almost daily.” The TAPS-2 is the brief assessment component (administered for any substance use endorsed in the TAPS-1) that asks participants 3-4 additional questions regarding use in the past 3 months. In total, the tool is comprised of 7-35 questions determined by a skip pattern. TAPS scores were coded as follows: For each substance, if TAPS-1= “never,” score = 0. For TAPS-1 ≥ 1, TAPS score = number of TAPS-2 items answered “yes.” Scores ranged from 0-4 for alcohol and 0-3 for all other substances. The cut-point of a score of ≥ 2 for tobacco/nicotine, alcohol, and cannabis were used to identify SUD.

© 2023 Levy S et al. *JAMA Network Open*.

eFigure 1. Description of Recruitment Methods

Virtual Recruitment
**Direct referral from primary care provider during a virtual healthcare visit:** Age-eligible patients at one of the PC sites presenting for a virtual health maintenance appointment were informed about the study during their visit and provided a videoconference link in the chat.
**Patient portal messaging:** All age eligible patients enrolled in one of the PC sites (N=36,989) received a message through their healthcare portal informing them of the study and inviting them to join a videoconference or leave their contact information for call back. Practices sent out messages between 3 and 5 times over the course of the study.
**Posters and post cards:** All participating PC and AM sites were provided wall posters and postcards with QR codes that directed adolescents to study contact information. Front desk staff and healthcare providers directed potentially eligible patients who presented for an in-person appointment to these resources.
**Phone calls to patients and parents/caregivers:** Age eligible patients scheduled for an appointment at AM and SUDTP were sent an IRB-approved letter to their home address or via email explaining the study and notifying them that an RA may contact them by phone and offering them an opportunity to "opt out" from future contact. At least one day after sending an electronic packet or one week after mailing a letter, the RA called the phone number listed on the patient's electronic medical record (EMR), which was either for the patient or the parent/guardian. Interested patients either 1) were sent a link to join videoconference session to begin the study immediately or 2) scheduled an appointment for a future videoconference. Additionally, SUDTP clinicians called each parent prior to their child's appointment as part of the clinical protocol and informed parents of the study, asked for a good time for the RA to reach them and recorded contact information.
In-person Recruitment
**Primary Care sites:** RAs were provided a list of age eligible patients' first names and appointment time the day prior to the scheduled appointment. On the day of recruitment, RAs were stationed in waiting rooms where they could approach eligible patients and, if interested, enroll them in the study. If time did not permit, patients were provided IRB-approved postcards with the study contact information and invited to complete the study via videoconference or share their contact information to receive a call back.
**AM:** An RA reviewed the clinic schedule to identify age eligible patients the day prior to in person recruitment and recorded new potential participants' information in the chart review log. On recruitment days, RAs approached participants in the waiting room before their visit or in the exam room prior to their visit to enroll interested patients. The RA completed as much of the study as possible before the appointment and could continue after the appointment as needed. If the participant did not have sufficient time to complete the study, they were provided IRB approved postcard with the study contact information and invited to complete the study over zoom.

Abbreviation: PC, community-based primary care pediatrics practices; RA, Research Assistant; SUDTP, outpatient adolescent substance use disorders program; AM, adolescent medicine program at a community pediatric practice affiliated with an academic institution.

© 2023 Levy S et al. *JAMA Network Open*.

eFigure 2. STARD Flow Diagram for Tobacco/Nicotine Use Disorder (TUD)[a]

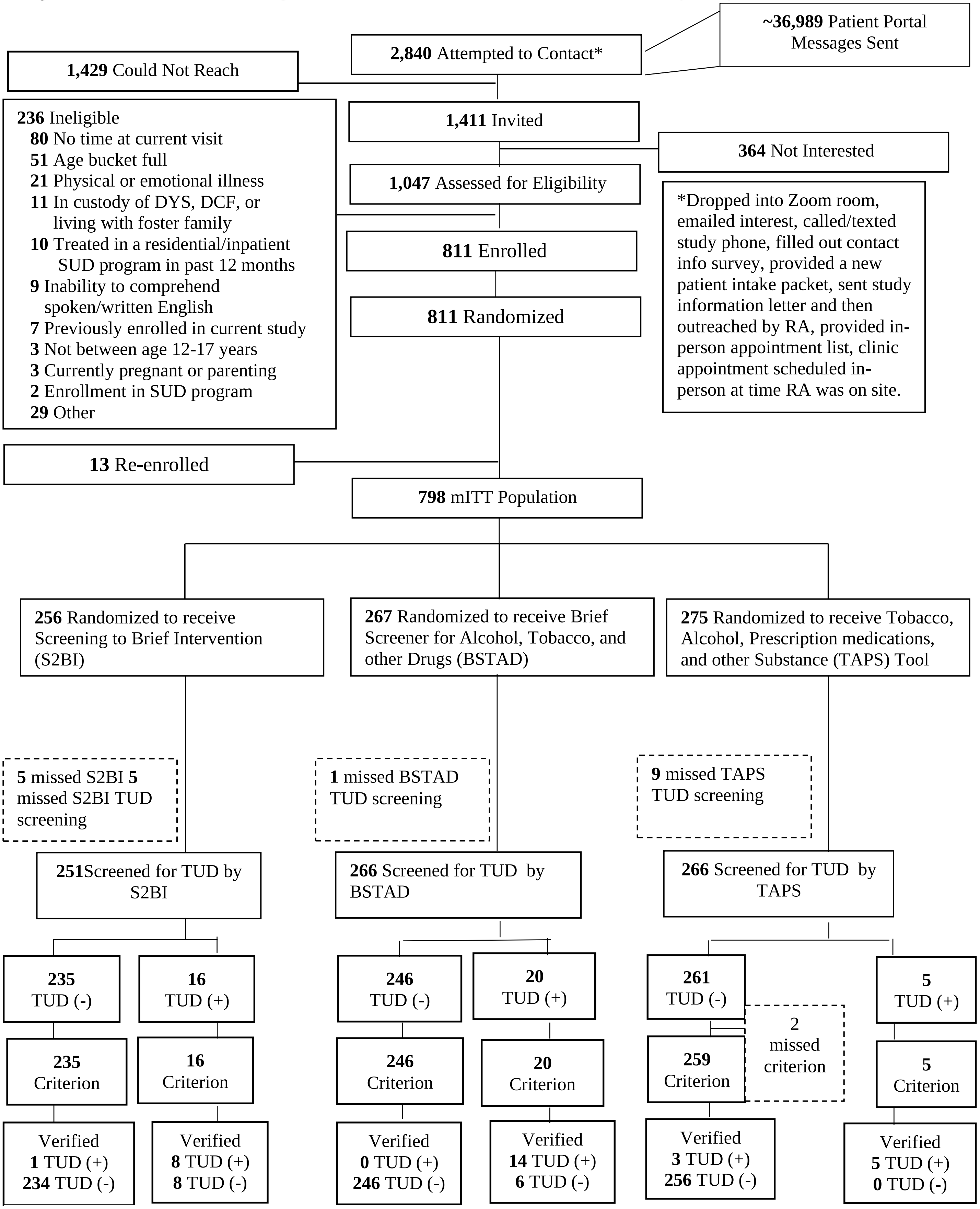

a. mITT: The modified intention to treat population consists of those who completed the study once and were used to analyze alcohol, cannabis, and tobacco use. All mITT participants were considered in the primary analysis but had to have both screening tool question(s) and CIDI score for a substance (tobacco/nicotine, alcohol, and cannabis) to be able to be used in calculating the psychometric properties.

© 2023 Levy S et al. *JAMA Network Open*.

eFigure 3. STARD Flow Diagram for Alcohol Use Disorder (AUD)[a]

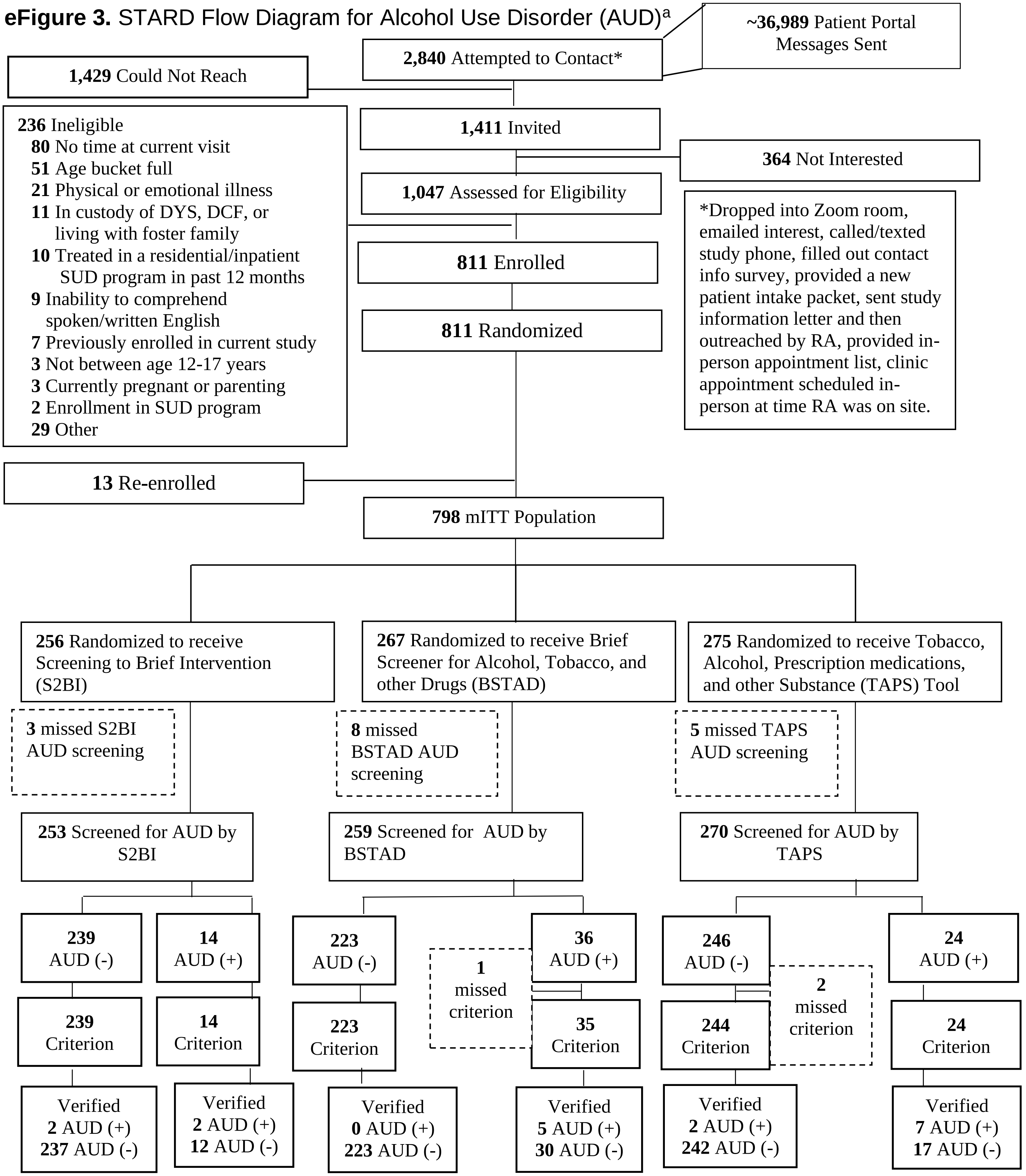

a. mITT: The modified intention to treat population consists of those who completed the study once and were used to analyze alcohol, cannabis, and tobacco use. All mITT participants were considered in the primary analysis but had to have both screening tool question(s) and CIDI score for a substance (tobacco/nicotine, alcohol, and cannabis) to be able to be used in calculating the psychometric properties.

© 2023 Levy S et al. *JAMA Network Open*.

eFigure 4. STARD Flow Diagram for Cannabis Use Disorder (CUD)[a]

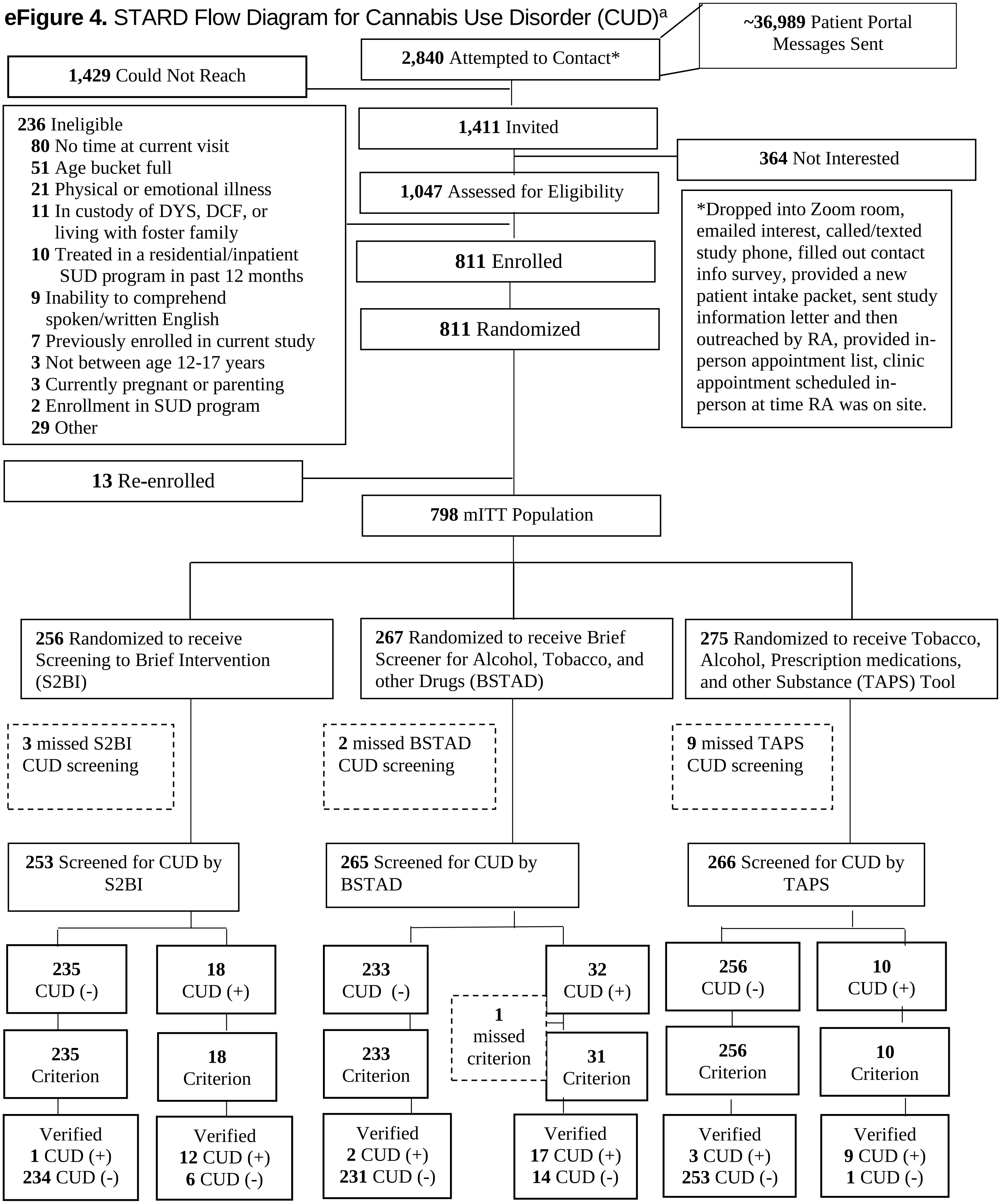

a. mITT: The modified intention to treat population consists of those who completed the study once and were used to analyze alcohol, cannabis, and tobacco use. All mITT participants were considered in the primary analysis but had to have both screening tool question(s) and CIDI score for a substance (tobacco/nicotine, alcohol, and cannabis) to be able to be used in calculating the psychometric properties.

© 2023 Levy S et al. *JAMA Network Open*.

eFigure 5. ROC Curves for S2BI, BSTAD, and TAPS Tool Screeners and Tobacco/Nicotine Use mITT Population

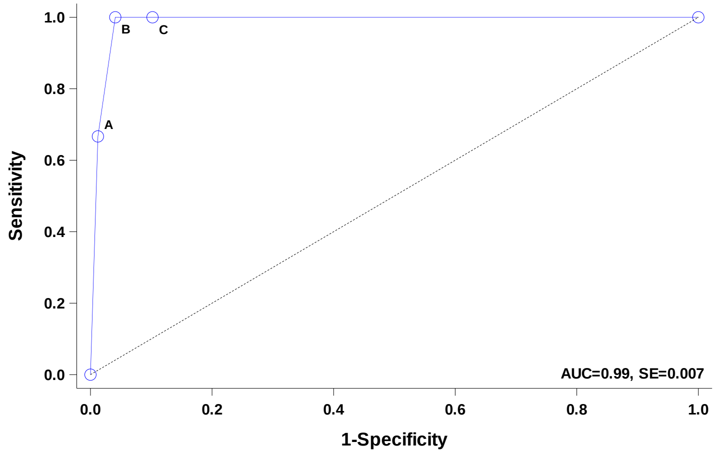

On the points in the scatter plot:
A represents S2BI Cut Point = 3 (> Monthly = 1; otherwise = 0),
B represents S2BI Cut Point = 2 (> Once or twice = 1; otherwise = 0),
C represents S2BI Cut Point = 1 (> Never = 1; otherwise = 0).

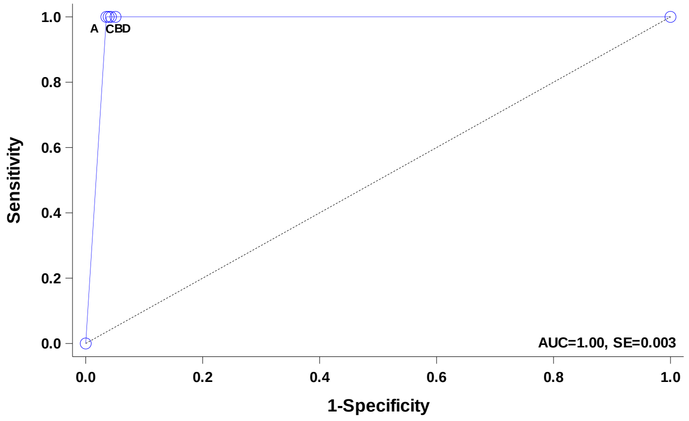

On the points in the scatter plot:
A represents BSTAD Cut Point = 4 (> 5 days = 1; otherwise = 0),
B represents BSTAD Cut Point = 3 (> 2 days = 1; otherwise = 0),
C represents BSTAD Cut Point = 2 (> 1 day = 1; otherwise = 0),
D represents BSTAD Cut Point = 1 (> 0 days = 1; otherwise = 0).

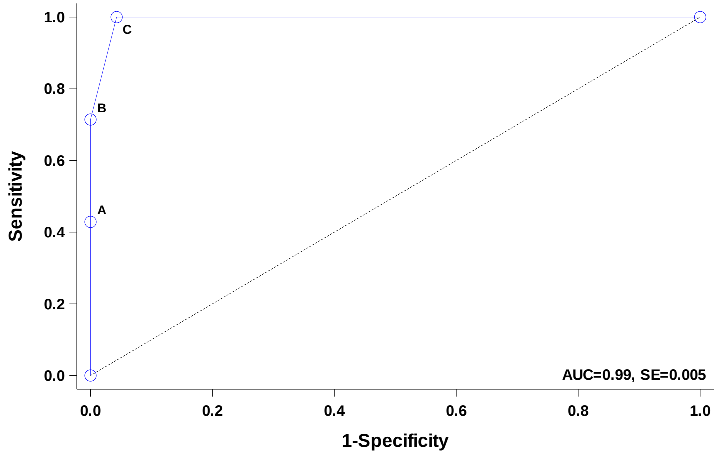

On the points in the scatter plot:
A represents TAPS Cut Point = 3 (>2 = 1; otherwise = 0),
B represents TAPS Cut Point = 2 (>1 = 1; otherwise = 0),
C represents TAPS Cut Point = 1 (>0 = 1; otherwise = 0).

© 2023 Levy S et al. *JAMA Network Open*.

eFigure 6. ROC Curves for S2BI, BSTAD, and TAPS Tool Screeners and Alcohol Use mITT Population

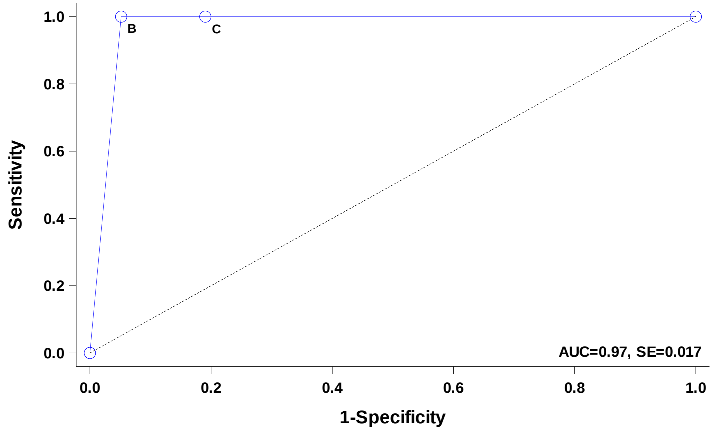

On the points in the scatter plot:
A represents S2BI Cut Point = 3 (> Monthly = 1; otherwise = 0),
B represents S2BI Cut Point = 2 (> Once or twice = 1; otherwise = 0),
C represents S2BI Cut Point = 1 (> Never = 1; otherwise = 0).
Point A is missing because no participants responded > monthly.

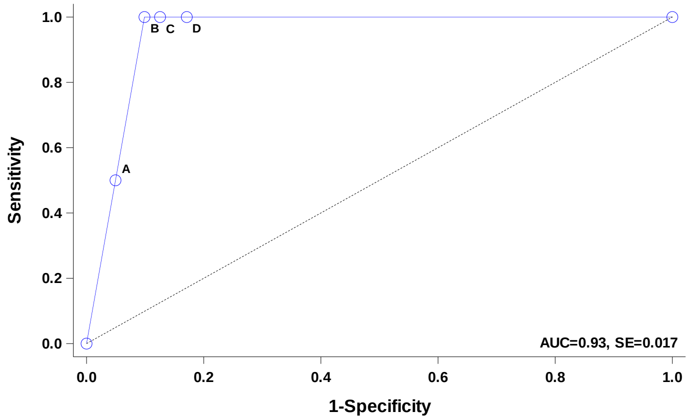

On the points in the scatter plot:
A represents BSTAD Cut Point = 4 (> 5 days = 1; otherwise = 0),
B represents BSTAD Cut Point = 3 (> 2 days = 1; otherwise = 0),
C represents BSTAD Cut Point = 2 (> 1 day = 1; otherwise = 0),
D represents BSTAD Cut Point = 1 (> 0 days = 1; otherwise = 0).

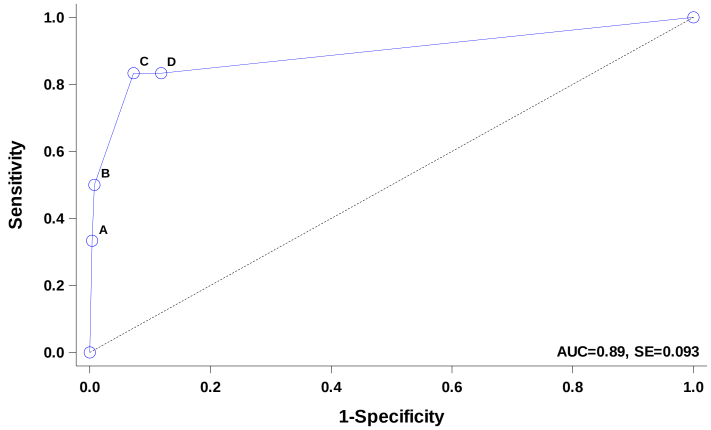

On the points in the scatter plot:
A represents TAPS Cut Point = 4 (>3 = 1; otherwise = 0),
B represents TAPS Cut Point = 3 (>2 = 1; otherwise = 0),
C represents TAPS Cut Point = 2 (>1 = 1; otherwise = 0),
D represents TAPS Cut Point = 1 (>0 = 1; otherwise = 0).

© 2023 Levy S et al. *JAMA Network Open*.

eFigure 7. ROC Curves for S2BI, BSTAD, and TAPS Tool Screeners and Cannabis Use mITT Population

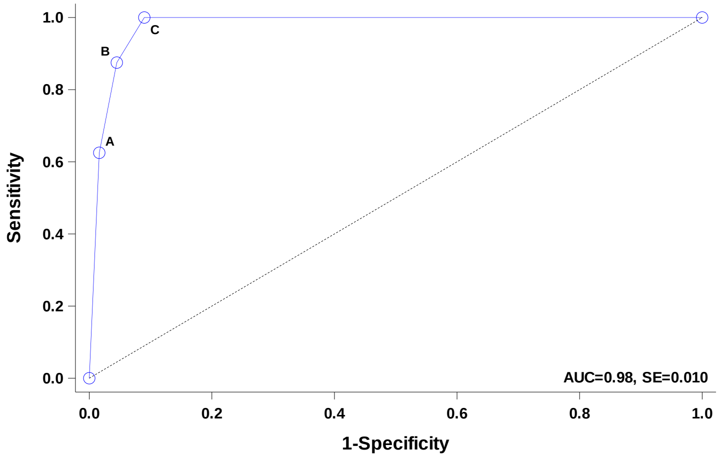

On the points in the scatter plot:
A represents S2BI Cut Point = 3 (> Monthly = 1; otherwise = 0),
B represents S2BI Cut Point = 2 (> Once or twice = 1; otherwise = 0),
C represents S2BI Cut Point = 1 (> Never = 1; otherwise = 0).

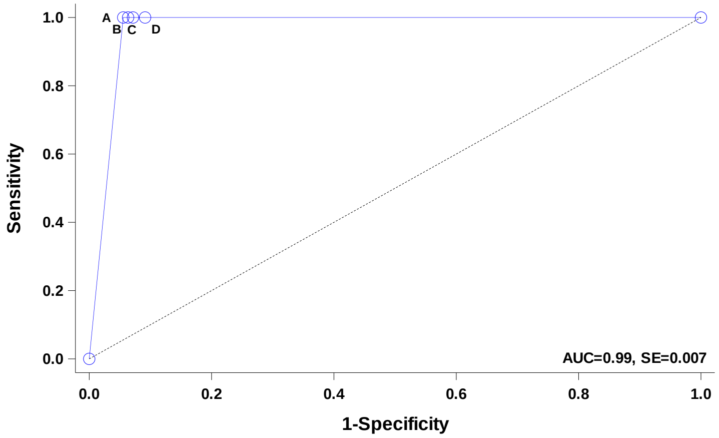

On the points in the scatter plot:
A represents BSTAD Cut Point = 4 (> 5 days = 1; otherwise = 0),
B represents BSTAD Cut Point = 3 (> 2 days = 1; otherwise = 0),
C represents BSTAD Cut Point = 2 (> 1 day = 1; otherwise = 0),
D represents BSTAD Cut Point = 1 (> 0 days = 1; otherwise = 0).

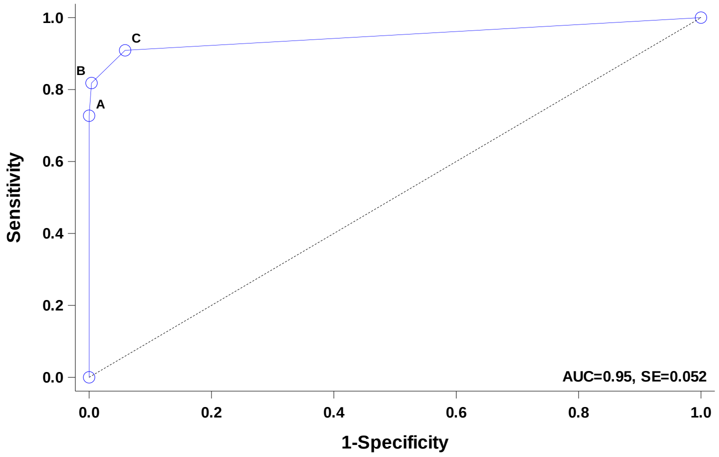

On the points in the scatter plot:
A represents TAPS Cut Point = 3 (>2 = 1; otherwise = 0),
B represents TAPS Cut Point = 2 (>1 = 1; otherwise = 0),
C represents TAPS Cut Point = 1 (>0 = 1; otherwise = 0).

© 2023 Levy S et al. *JAMA Network Open*.

eTable 1. Prevalence of Substance Use Disorder on the Criterion Measure (CIDI) Among mITT Participants (N=798)

**No. (%)**	**Criterion : CIDI Total *(N = 798)***
**Tobacco/Nicotine**	
Substance Use Disorder	32 (4.0)
Use without SUD	16 (2.0)
No use	741 (92.9)
Missing	9 (1.1)
**Alcohol**	
Substance Use Disorder	18 (2.3)
Use without SUD	22 (2.8)
No use	748 (93.7)
Missing	10 (1.3)
**Cannabis**	
Substance Use Disorder	44 (5.5)
Use without SUD	13 (1.6)
No use	731 (91.6)
Missing	10 (1.3)
**Opiates**	
Substance Use Disorder	1 (0.1)
Use without SUD	1 (0.1)
No use	787 (98.6)
Missing	9 (1.1)
**Benzodiazepines**	
Substance Use Disorder	4 (0.5)
Use without SUD	0 (0.0)
No use	785 (98.4)
Missing	9 (1.1)

© 2023 Levy S et al. *JAMA Network Open*.

eTable 2. Prevalence of Substance Use Disorders by Study Site, Based on the Criterion (CIDI) Among mITT Participants (N=798)

No. (%)	*Total* 798	*Sites* *SUDTP* [d] 41 (5.1)	*AM* [e] 84 (10.5)	*PC* [f] 673 (84.3)	*P*
**Tobacco/Nicotine** [a]					
Substance Use Disorder	32 (4.0%)	22 (53.7%)	2 (2.4%)	8 (1.2%)	<0.001***
**Alcohol** [b]					
Substance Use Disorder	18 (2.3%)	11 (26.8%)	0 (0.0%)	7 (1.0%)	<0.001***
**Cannabis** [c]					
Substance Use Disorder	44 (5.5%)	31 (75.6%)	5 (6.0%)	8 (1.2%)	<0.001***

Column percentages are shown.

a. Denominator includes n=9 who did not complete the study.

b. Denominator includes n=10 who did not complete the study.

c. Denominator includes n=10 who did not complete the study.

d. An outpatient adolescent SUD treatment program at a pediatric hospital (SUDTP)

e. The adolescent medicine program at a community-based pediatrics practice serving primarily low-income patients (AM)

f. Participating pediatric primary care practices (PC)

© 2023 Levy S et al. *JAMA Network Open*.

eTable 3. Disclosure of Other Past 12-Month Substance Use Among the Participants Randomized to S2BI/BSTAD Screeners (n=523)

		Screener tool	
**No. (%)**	**Total**	**S2BI[a]**	**BSTAD[b]**
		256 (48.9)	267 (51.1)
**Past 12 month Other Substance Use [c]**			
Any	37 (7.1)	25 (9.8)	12 (4.5)
Never	478 (91.4)	225 (87.9)	253 (94.8)
Missing	8 (1.5)	6 (2.3)	2 (0.7)
**Past 12 month Prescription Medications Use**			
Any	31 (5.9)	21 (8.2)	10 (3.7)
Never	486 (92.9)	230 (89.8)	256 (95.9)
Missing	6 (1.1)	5 (2.0)	1 (0.4)
**Past 12 month Illegal Drugs Use**			
Any	5 (1.0)	1 (0.4)	4 (1.5)
Never	513 (98.1)	252 (98.4)	261 (97.8)
Missing	5 (1.0)	3 (1.2)	2 (0.7)
**Past 12 month Inhalants (S2BI cohort only) Use**			
Any	0 (0.0)	0 (0.0)	
Never	253 (98.8)	253 (98.8)	
Missing	3 (1.2)	3 (1.2)	
**Past 12 month Herbs or Synthetic Drugs (S2BI cohort only) Use**			
Any	5 (2.0)	5 (2.0)	
Never	247 (96.5)	247 (96.5)	
Missing	4 (1.6)	4 (1.6)	

Column percentages are shown.

a. S2BI assesses the past 12-month use of tobacco, alcohol, cannabis, prescription medication, e-cigarette, illegal drugs, inhalants, and herbs or synthetic drugs. Results for other substance than tobacco/e-cigarette, alcohol or cannabis are reported in this table.

b. BSTAD assesses the past 12-month use of nicotine/tobacco, alcohol, cannabis, prescription medications and illegal drugs. Results other than nicotine/tobacco, alcohol, or cannabis are reported in this table.

c. Past 12-month use of any other substance than tobacco/nicotine, alcohol or cannabis.

© 2023 Levy S et al. *JAMA Network Open*.

eTable 4. Disclosure of Any Past 3-Month Substance Use Among the Participants Randomized to the TAPS Screener (n=275)

No. (%)	TAPS [a] 275 (100)
**TAPS Screener 1**	
**Past 12 month Prescription Medications Use**	
Any	14 (5.1)
Never	255 (92.7)
Missing	6 (2.2)
**Past 12 month Illegal Drugs Use**	
Any	32(11.6)
Never	234 (85.1)
Missing	9 (3.3)
**TAPS Screener 2 [b]**	
**Past 3 month Prescription Cannabis Use**	
Any	25 (9.1)
Never	241 (87.6)
Missing	9 (3.3)
**Past 3 month Prescription Opiate Pain Reliever Use**	
Any	1 (0.4)
Never	268 (97.5)
Missing	6 (2.2)
**Past 3 month Heroin Use**	
Any	0 (0.0)
Never	266 (96.7)
Missing	9 (3.3)
**Past 3 month Medication for Anxiety or Sleep Use**	
Any	2 (0.7)
Never	267 (97.1)
Missing	6 (2.2)
**Past 3 month Medication for ADHD Use**	
Any	1 (0.4)
Never	268 (97.5)
Missing	6 (2.2)
**Past 3 month Cocaine, Crack, or Methamphetamine Use**	
Any	0 (0.0)
Never	266 (96.7)
Missing	9 (3.3)
**Past 3 month Other Drugs Use**	
Any	2 (0.7)
Never	255 (92.7)
Missing	18 (6.5)
**Past 3 month Other Drugs, specified:**	
"Codeine, shrooms"	1 (0.4)
"None"	1 (0.4)

Column percentages are shown.

© 2023 Levy S et al. *JAMA Network Open*.

a. The TAPS Tool is comprised of TAPS-1 which asks 1 item on frequency of use for each substance in past 12 months using 5 options ranging from never to daily or almost daily, and TAPS-2 which asks 3 or 4 yes/no questions about use in the past 3 months.

b. Past 3-month questions were asked among those who positively responded to the TAPS-1 questions.

eTable 5. Performance of S2BI, BSTAD, and TAPS Tool Screeners vs CIDI for Identifying Substance Use Disorder, Excluding SUDTP Population

	True Positive (n)	False Positive (n)	True Negative (n)	False Negative (n)	Sensitivity (95% CI)	Specificity (95% CI)	PPV (95% CI)	NPV (95% CI)	Positive LR (95% CI)	Negative LR (95% CI)
**S2BI (cutoff ≥ monthly)**										
**Risk Level: Substance use disorder (CIDI 2+)**										
Tobacco/Nicotine	2	4	231	1	0.67 (0.09, 0.99)	0.98 (0.96, 1.00)	0.33 (0.04, 0.78)	1.00 (0.98, 1.00)	39.17 (11.12, 137.90)	0.34 (0.07, 1.68)
Alcohol	1	9	228	2	0.33 (0.01, 0.91)	0.96 (0.93, 0.98)	0.10 (0.00, 0.45)	0.99 (0.97, 1.00)	8.78 (1.57, 49.21)	0.69 (0.31, 1.54)
Marijuana	5	4	231	0	1.00 (0.48, 1.00)	0.98 (0.96, 1.00)	0.56 (0.21, 0.86)	1.00 (0.98, 1.00)	58.75 (22.23, 155.23)	0[a] (-)
**BSTAD (cutoff ≥2)**										
**Risk Level: Substance use disorder (CIDI 2+)**										
Tobacco/Nicotine	5	3	244	0	1.00 (0.48, 1.00)	0.99 (0.96, 1.00)	0.63 (0.24, 0.91)	1.00 (0.98, 1.00)	82.33 (26.74, 253.53)	0[a] (-)
Alcohol	1	23	220	0	1.00 (0.03, 1.00)	0.91 (0.86, 0.94)	0.04 (0.00, 0.21)	1.00 (0.98, 1.00)	10.57 (7.16, 15.59)	0[a] (-)
Marijuana	4	13	231	2	0.67 (0.22, 0.96)	0.95 (0.91, 0.97)	0.24 (0.07, 0.50)	0.99 (0.97, 1.00)	12.51 (5.77, 27.15)	0.35 (0.11, 1.09)
**TAPS Tool (cutoff ≥2)**										
**Risk Level: Substance use disorder (CIDI 2+)**										
Tobacco/Nicotine	1	0	251	1	0.50 (0.01, 0.99)	1.00 (0.99, 1.00)	1.00 (0.03, 1.00)	1.00 (0.98, 1.00)	0[a] (-)	0.50 (0.13, 2.00)
Alcohol	3	13	240	0	1.00 (0.29, 1.00)	0.95 (0.91, 0.97)	0.19 (0.04, 0.46)	1.00 (0.98, 1.00)	19.46 (11.46, 33.05)	0[a] (-)
Marijuana	1	1	251	1	0.50 (0.01, 0.99)	1.00 (0.98, 1.00)	0.50 (0.01, 0.99)	1.00 (0.98, 1.00)	126.00 (11.46, 1385.21)	0.50 (0.13, 2.01)

PPV = Positive Predictive Value; NPV = Negative Predictive Value; CI = Confidence Interval; LR = Likelihood Ratio.

a. Estimates and confidence intervals not able to be calculated due to 0 counts or division by 0 are denoted with "-"in the table.

© 2023 Levy S et al. *JAMA Network Open*.

eReferences

1. Levy S, Weiss R, Sherritt L, et al. An Electronic Screen for Triaging Adolescent Substance Use by Risk Levels. *JAMA Pediatr*. 2014;168(9):822-828. doi:10.1001/jamapediatrics.2014.774
2. Levy S, Weitzman ER, Marin AC, Magane KM, Wisk LE, Shrier LA. Sensitivity and specificity of S2BI for identifying alcohol and cannabis use disorders among adolescents presenting for primary care. *Subst Abus*. 2021;42(3):388-395. doi:10.1080/08897077.2020.1803180
3. Kelly SM, Gryczynski J, Mitchell SG, Kirk A, O'Grady KE, Schwartz RP. Validity of Brief Screening Instrument for Adolescent Tobacco, Alcohol, and Drug Use. *Pediatrics*. 2014;133(5):819-826. doi:10.1542/peds.2013-2346
4. Wu LT, McNeely J, Subramaniam GA, Sharma G, VanVeldhuisen P, Schwartz RP. Design of the NIDA clinical trials network validation study of tobacco, alcohol, prescription medications, and substance use/misuse (TAPS) tool. *Contemp Clin Trials*. 2016;50:90-97. doi:10.1016/j.cct.2016.07.013

© 2023 Levy S et al. *JAMA Network Open*.
