# Supplementary material for: Assessment of Screening Tools to Identify Substance Use Disorders Among Adolescents
Source: JAMA Netw Open. 2023 May 22;6(5):e2314422. doi: 10.1001/jamanetworkopen.2023.14422 (PMC10203888; doi:10.1001/jamanetworkopen.2023.14422)
Supplement: Supplement 2. — Data Sharing Statement [file jamanetwopen-e2314422-s002.pdf]

Data Sharing Statement

Levy. Assessment of Screening Tools to Identify Substance Use Disorders Among Adolescents. *JAMA Netw Open*. Published May 22, 2023.
doi:10.1001/jamanetworkopen.2023.14422

Data
Data available: Yes
Data types: Deidentified participant data
How to access data: https://datashare.nida.nih.gov/data
When available: With publication

Supporting Documents
Document types: None

Additional Information
Who can access the data: Data will be publicly available.
Types of analyses: Any purpose
Mechanisms of data availability: without investigator support
